# The dynamics of apoplast phenolics in tobacco leaves following inoculation with bacteria

**DOI:** 10.3389/fpls.2015.00649

**Published:** 2015-08-20

**Authors:** Con J. Baker, Norton M. Mock, Jodi M. Smith, Andrey A. Aver'yanov

**Affiliations:** ^1^Molecular Plant Pathology Lab., U.S. Department of AgricultureBeltsville, MD, USA; ^2^All-Russian Research Institute of Phytopathology, Russian Agricultural AcademyMoscow, Russia

**Keywords:** *pseudomonas fluorescens*, syringae, chlorogenic acid, acetosyringone, acetovanillone, *tabaci*, acetophenones

## Abstract

This study demonstrates that the accumulation of apoplastic phenolics is stimulated *in planta* in response to bacterial inoculation. Past studies have shown that levels of extracellular phenolics are elicited in plant cell suspensions in response to bacteria, and that tomato plants infected with viroids showed changes in apoplastic phenolics. The method described here monitored changes in apoplastic phenolics in tobacco leaves following bacterial inoculation of the same tissue. Inoculation with a saprophyte, *Pseudomonas fluorescens*, which does not cause visible symptoms or physical damage, was used to elicit phenolics and examine the effects of variable parameters on phenolic composition. Location of the inoculation on the leaf, position, or developmental age of the leaf on the plant, and inoculum concentration were standardized for further experiments. The patterns of phenolic change in the apoplast were compared for tobacco inoculated with *P. syringae* pathovars, pv. *syringae*, which causes a resistant HR reaction within 15 h, and pv. *tabaci*, which causes a susceptible reaction with delayed visible symptoms. Both pathogens elicited lower increased levels of acetosyringone compared to the saprophyte, *P. fluorescens* but had greatly increased levels of the chlorogenic acid derivatives. The latter metabolites appear to have come from the intracellular stores, which could indicate a weakening of the apoplast/symplast barrier. This unexpected aspect will require further study of intracellular phenolics.

## Introduction

In regard to disease resistance, the plant apoplast, which surrounds the plant cell, is analogous to a moat protecting a castle within. The tactics used by both the host and pathogen on this battle ground, outside the protected cell, can be complex, having evolved over time, and can create very toxic conditions and molecules (Vanacker et al., 1998; Bolwell et al., 2001; Pechanova et al., 2010; Martínez and Guiamet, 2014). Only pathogens that have evolved adequate counter measures to slip through the apoplast will survive and cause disease. In regard to disease control, the leaf apoplast is highly vulnerable to manipulation either by genetic engineering or direct application of materials to leaves, via stomata or additions to ground water, and delivered by xylem. By knowing how pathogen and host tactics operate in the apoplast we may be able to enhance specific attributes and better protect the cell within from pathogens and other stressors.

Our research has focused on the redox/phenolic events that change dramatically in the apoplast following pathogen inoculation. Most of these previous studies were carried out using cell suspensions of potato and tobacco that were inoculated with pathovars of *Pseudomonas syringae*. Each cell suspension line produced a unique set of extracellular phenolics consisting of hydroxycinnamic acid derivatives as well as amine conjugates (Baker et al., 2005b, 2009). Upon inoculation with different bacterial species or elicitors, the composition of these extracellular phenolics would change within hours. For example, when tobacco cell suspensions were inoculated with *P. syringae* pv. *syringae*, an incompatible pathovar that causes a hypersensitive response on plant leaves, the extracellular concentration of acetosyringone increased compared to controls until an oxidative burst occurred several hours later. The acetosyringone disappeared concurrent with the oxidative burst, and its oxidized product likely contributed to the subsequent increase in redox potential (Baker et al., 2005a, 2013, 2014). In potato cell suspensions the differential induction of extracellular phenolics by bacteria or elicitors was very apparent with the amide phenolics, feruloyloctopamine (FOA), and coumaroyloctopamine (COA) (Baker et al., 2009). Potato suspensions treated with a fungal extract induced a 20-fold increase in COA after 6 h, while suspensions inoculated with *P. s*. pv. *syringae*, which is incompatible and causes an oxidative burst, reduced the amount of FOA nearly 20-fold. These studies demonstrated that pathogens or related products caused rapid changes in the extracellular phenolics of cell suspensions and the specifics of the change varied with the pathogen or elicitor.

In a more recent study with greenhouse tomato plants (Baker et al., 2010), we demonstrated that the apoplast phenolics of whole plants also respond to infection. Plants were inoculated with the pathogen, *Potato spindle tuber viroid* (PSTVd), which remains in the symplast spreading cell to cell via plasmodesmata throughout the plant. By examining the apoplastic phenolics of leaves on plants at varying stages of infection, we found changes in apoplastic phenolics as the viroid spread throughout the plant over several weeks. This study demonstrated that whole plants, respond to pathogens with changes in apoplastic phenolics, similar to the cell suspension studies.

Using the methods developed for analyzing apoplastic phenolics in tomato leaves (Baker et al., 2010, 2012), we wanted to examine the dynamics of apoplastic phenolics in tobacco leaves upon inoculation with bacteria. The objective was to determine if there was a unique dynamic for saprophytic, virulent, and avirulent interactions, as we had found in cell suspension studies (Baker et al., 2005b, 2009). A major technological concern was whether the tissue that had been inoculated with bacteria would be amenable to the water-infiltration used for subsequent analyses. Would the bacteria cause damage or would bacterial polysaccharides block the interstices of the apoplast and prevent representative sampling of the phenolics? Several parameters that were found to impact changes in apoplast composition were examined and standardized. An unexpected result suggests that this technique may also indicate how different bacterial/plant interactions (saprophytic, virulent, avirulent) affect the plant apoplast/symplast boundary.

## Materials and methods

### Chemicals

All chemicals were obtained from Sigma-Aldrich Chemicals, Inc (St. Louis, MO). Standard solutions, 50 μM, were prepared in deionized water for acetosyringone (3′,5′-dimethoxy-4′-hydroxyacetophenone, ACE), acetovanillone (3′-methoxy-4′-hydroxyacetophenone, AV), chlorogenic acid (3-O-caffeoylquinic acid, CGA-A), neochlorogenic acid (trans-5-O-caffeoylquinic acid, CGA-B), 4-O-caffeoylquinic acid (CGA-C).

### Preparation of bacteria

*Pseudomonas syringae* pv *syringae* (PSS), *P. s*. pv. *tabaci* (PTAB), and *P. fluorescens* (PF) were routinely maintained on King's B media; augmented with 25 μg/ml naladixic acid for PSS. PSS caused tissue collapse and a resistant hypersensitive response (HR) on tobacco after 15 h, while PTAB caused a susceptible response and developed symptoms more slowly; PF is considered a non-pathogen causing no visible symptoms. Long-term storage of the bacteria was in the same media supplemented with 15% glycerol and stored in liquid nitrogen. For experiments, bacteria were grown for 18–20 h in King's B broth (augmented with naladixic acid for PSS) in dark, at 190 rpm, 30 C. Overnight cultures were centrifuged at 8000 rpm for 3 min at room temperature in a fixed rotor. Pellets were washed with deionized water, centrifuged, and then suspended in deionized water to various concentrations, based on optical density; 0.1 ODU _600nm_ was about 10^8^ CFU ml^−1^.

### Preparation of plant material

Tobacco plants (*Nicotiana tabacum*) *cv* Hicks were grown under greenhouse conditions at 21–27 C with a 14 h photoperiod supplemented by High Intensity Discharge lamps. Plants were seeded in Sunshine Professional Growing Mix #5/LP5 (Sun Gro Horticulture Distribution Inc., Agawam, MA) and transplanted 2 weeks later into 2 inch pots. Plants were transplanted again after another 2 weeks into 4 inch pots. At 1–2 weeks later, plants were transferred to 6 inch pots containing 50/50 Sunshine Professional Growing Mix #5/LP5 and Sunshine Professional Growing Mix #1/LC1 (Sun Gro Horticulture Distribution Inc., Agawam, MA). When plants were transplanted, Scotts Osmocote Plus (15-9-12) Controlled Release Fertilizer (The Scotts Miracle-Gro Company, Marysville, OH) was added to the potting mix. Plants were routinely used for experiments at approximately 6 weeks of age, having 7–10 fully developed leaves.

Leaves were numbered from the top down starting with the first fully developed leaf. This procedure helped insure that leaves of the same number were similar in developmental age. Leaves 3 and 4 were generally used and inoculated with 10^8^ CFU ml^−1^, filling the panels in the middle of the leaf. This was carried out by pricking the leaf panel in a few spots and then gently pressing a plastic needle-less syringe barrel, filled with inoculum, against the leaf, which is supported on the opposite side by hand. To avoid possible contamination from one segment to another only one treatment per leaf was used on the 3rd and 4th leaf of the same plant. Two to three plants were used per experiment and all experiments were repeated at least two times with 2–4 replicates of each bacterial treatment.

### Extraction of apoplast phenolics

The apoplast phenolics from treated leaves were obtained by water infiltration followed by centrifugation as described previously for tomato leaves (Baker et al., 2012). Plants were watered 30 min prior to extraction to help ensure that all plants were of similar turgor. Keeping treatments and replications separate, each leaf was removed from the plant and rinsed with deionized water to remove contaminates from the leaf surface/trichomes. Panels, between secondary veins, were cut from the leaf and weighed immediately, about 0.5–0.8 g. Vacuum infiltration of the leaflets was carried out in a 500 ml side-arm flask containing 150 ml deionized water. The vacuum was released and reapplied successively 4–8 times in an attempt to reach 90–100% infiltration, which was estimated by the dark green color of the saturated tissue. Following infiltration the leaf panels were dried lightly with kimwipes, and post-infiltration weight was taken. To collect apoplast wash fluid (AWF), the leaf panels for each treatment/replication were stacked individually between sheets of parafilm (Pechiney Packaging, Chicago IL) and rolled around a 2 ml pipet tip (for support, pipette tip up) and inserted into a 30 ml syringe (with tip cut at an angle to allow drainage). The syringe was inserted into a 50 ml polycarbonate centrifuge tube. The tissue was centrifuged at 1800 rpm (600 g) for 10 min at 24°C using a Sorvall SH3000 swinging bucket rotor. The AWF that collected in the centrifuge tube was weighed and about 200 μl placed in a tapered 0.350 ml glass vial with 2 μl of 10% phosphoric acid to acidify the sample and help stabilize it until analysis by UPLC/MS as described below.

### Analysis of phenolics by UPLC/UV/MS

Phenolic analysis was conducted using an ultra pressure liquid chromatography system (Waters Acquity UPLC, Milford, MA, USA) equipped with a refrigerated autosampler, a photodiode array detector (PDA), and ESI interface and single-quadrupole MS detector (Thermo Fisher Scientific, Waltham, MA, USA). Samples, 4 μl, were separated on an Acquity BEH C18 (2.1 × 50 mm, 1.7 μm) column using a binary solvent system of 0.03% formic acid and methanol at a flow rate of 0.15 ml per minute. The gradient started with 10% methanol for 0.5 min followed by a linear increase to 40% by 12.7 min, then an increase to 90% within 2.3 min, a 3 min decline to 10%, and a 2 min hold at 10% methanol. The PDA measured absorbance from 210 to 400 nm. Peaks were identified by their retention times and spectra, and quantified by the peak height at the wavelength of maximum absorbance. Mass spectrometer parameters were: ESI probe temperature of 350 C, scan time 0.25 s and cone voltage of 50 V. Molecular weight or fragment masses (determined by mass spectrometer) were used to verify comparison of peaks between samples. Standard compounds were also injected onto C18 UPLC column and analyzed using the same conditions.

## Results

### Characterization of parameters affecting phenolic induction

Since the purpose of this study was to examine bacteria-induced phenolics, *P. fluorescens* (PF), a saprophyte commonly used as a point of reference in studies of pseudomonas pathogenicity (Oh et al., 2010), was used for method development. Previous studies with tobacco cell suspensions had demonstrated that PF treatment induced high levels of extracellular phenolics (Baker and Mock, 2004); also it had the advantage of not causing symptoms or physical damage to the tobacco leaf.

#### Effect of segment location within the leaf

The Hicks' mature tobacco leaf is relatively large, often 30–35 cm in length, and is divided in half by a prominent mid-vein. Each leaf half is segmented from the base to the tip by secondary veins, which also serve to limit infiltrated liquid to each segment. Leaf segments were infiltrated with PF, 10^8^ CFU ml^−1^, and after 6 h, the infiltrated segments were cut from the leaf and subjected to vacuum infiltration with water followed by centrifugation for analysis by UPLC/UV/MS as described in Materials and Methods.

**Figure 1:**
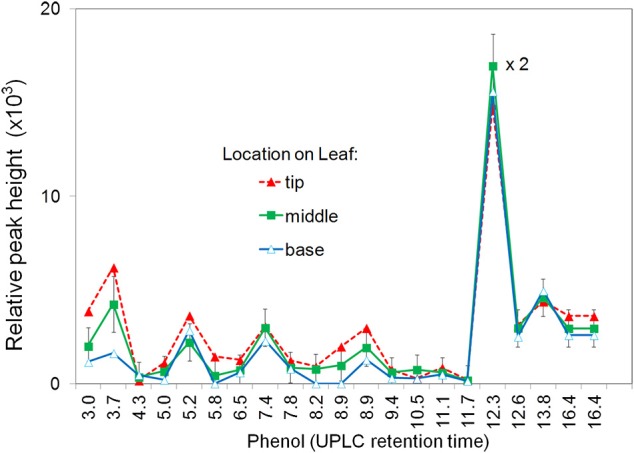
**Effect of segment location within the leaf on production of apoplastic phenolics in response to ***P. fluorescens*****. Leaf segments located at the tip, middle, or base of the leaf were infiltrated with 10^8^ CFU ml^−1^ *P. fluorescens*. After 6 h the segments were analyzed for phenolic content using UPLC/UV/MS. The major phenolics are identified by retention time. See Materials and Methods for further details.

The induced apoplastic phenolics from base, center, and tip segments of the same leaf were compared (Figure 1). The same phenolics, were found in all segments with a slight increase in relative concentration from base to tip segments. For further experiments only the two center segments on either side of midrib were used. To avoid possible contamination from one segment to another only one treatment per leaf was used.

#### Effect of leaf age

**Figure 2:**
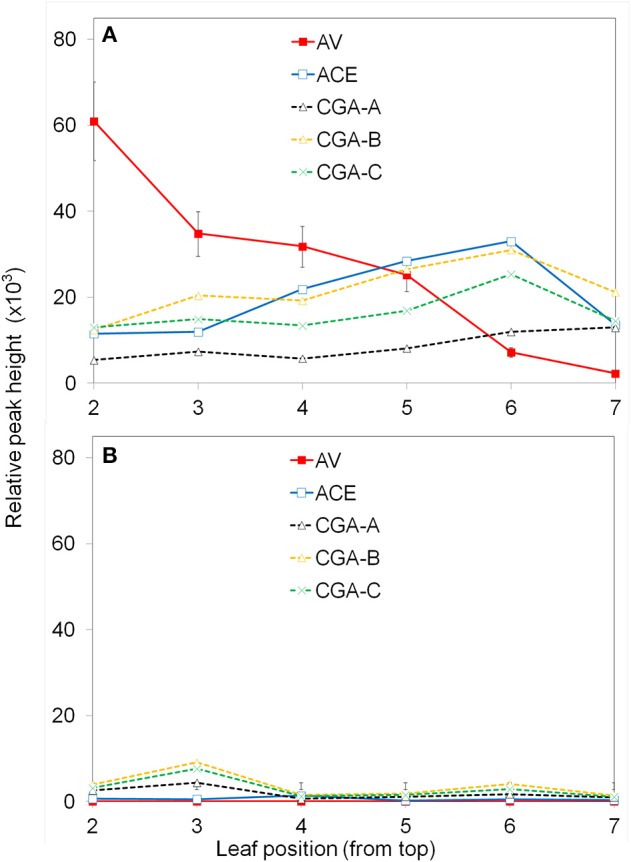
**Effect of leaf age on production of apoplastic phenolics in response to ***P. fluorescens*****. Leaves were numbered from the top down as described in the Materials and Methods. The leaf segments were inoculated with 10^8^ CFU ml^−1^ *P. fluorescens* **(A)**; control segments were inoculated with water **(B)**. After 6 h the segments were analyzed for phenolic content using UPLC/UV/MS. See Materials and Methods for further details; AV (acetovanillone); ACE (acetosyringone); CGA (chlorogenic acid isomers).

To determine the change of apoplastic phenolics with leaf age, all leaves from 5 and 7-week old and plants were compared. The first leaf that appeared fully or nearly fully expanded was number one and the numbering continued down the stem. The center two segments of each leaf was inoculated with PF, 10^8^ CFU ml^−1^, and the induced phenolics analyzed after 6 h (Figure 2A). Each leaf of another plant was inoculated with water as a control (Figure 2B).

Only certain major phenolics which were found to be of interest throughout the study, are shown (Figure 2). Acetovanillone (AV) and acetosyringone (ACE) are hydroxycinnamic acids. Three chlorogenic acid isomers designated CGA−A, −B, −C were found to co-elute with 5-O-Caffeoylquinic acid; 3-O-caffeoylquinic acid; and 4-O-caffeoylquinic acid, respectively. The exact structures of the CGA isomers are not confirmed at this time. PF clearly induced higher levels of these phenolics and the amount varied with leaf position. The concentration of induced acetovanillone was much higher in upper leaves and decreased in lower leaves. The other induced phenolics stayed about the same. The experiment was repeated twice with similar results. For further experiments, the 3rd and 4th leaves on similar plants of the same age were used.

#### Effect of inoculum concentration

**Figure 3:**
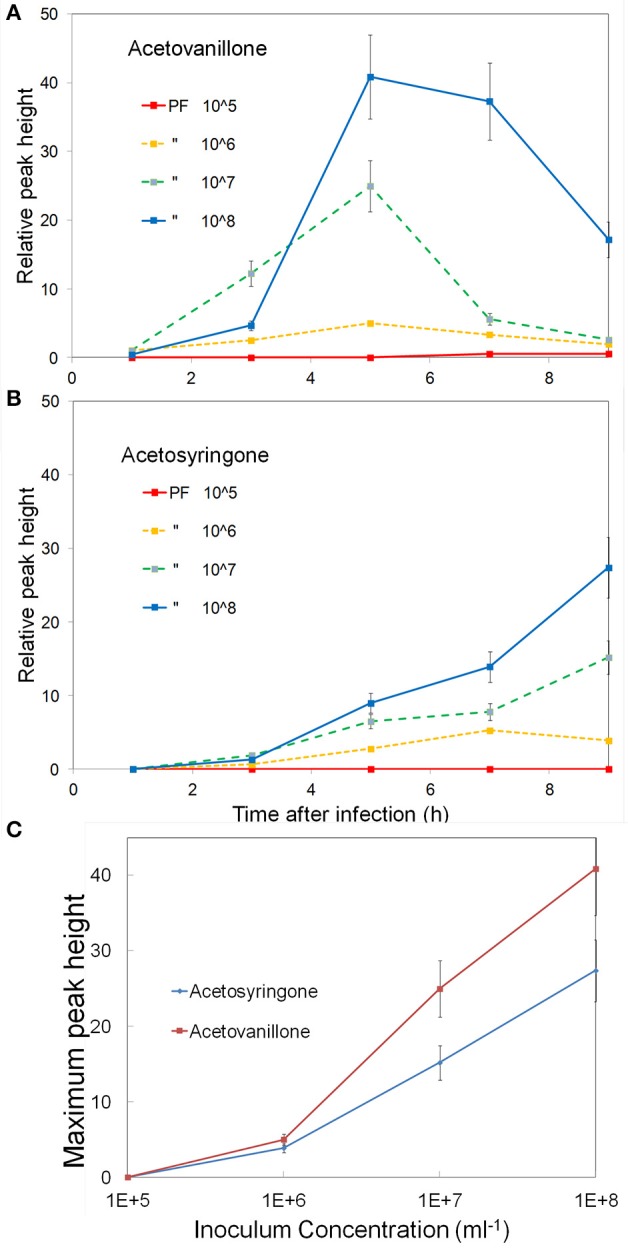
**Effect of inoculum concentration on the induction of apoplastic phenolics**. Tobacco leaf segments were inoculated with varying concentrations of *P. fluorescens*, 10^5^–10^8^ CFU ml^−1^, as indicated on the figure. The relative concentrations of two phenolics are shown over a 9 h period, **(A)** Acetovanillone and **(B)** Acetosyringone. **(C)** The peak concentrations of these metabolites vs. the log of inoculum concentration is shown. See Materials and Methods for further details.

Studies with cell suspensions indicated that the concentration of induced phenolics increased with inoculum concentration (Baker et al., 2005b). To examine this in leaves we varied the inoculum concentration from 10^5^ to 10^8^ CFU ml^−1^ and followed the induced phenolics over a 9 h period. The concentration of induced acetovanillone and acetosyringone are shown in (Figures 3A,B). The rate of induction of the phenolics increased with inoculum concentration (Figure 3C). Acetovanillone induction peaked at about 5 h for 10^6^–10^8^ CFU ml^−1^ suggesting the induction period was independent of inoculum concentration (Figure 3A), while acetosyringone continues to increase over this time period (Figure 3B). The experiment was repeated twice with similar results. For further experiments 10^8^ CFU ml^−1^ was routinely used for all bacteria.

### Time courses monitoring induced apoplast phenolics in saprophytic, virulent, and avirulent bacteria/tobacco interactions

#### *P. fluorescens*, a saprophyte

**Figure 4:**
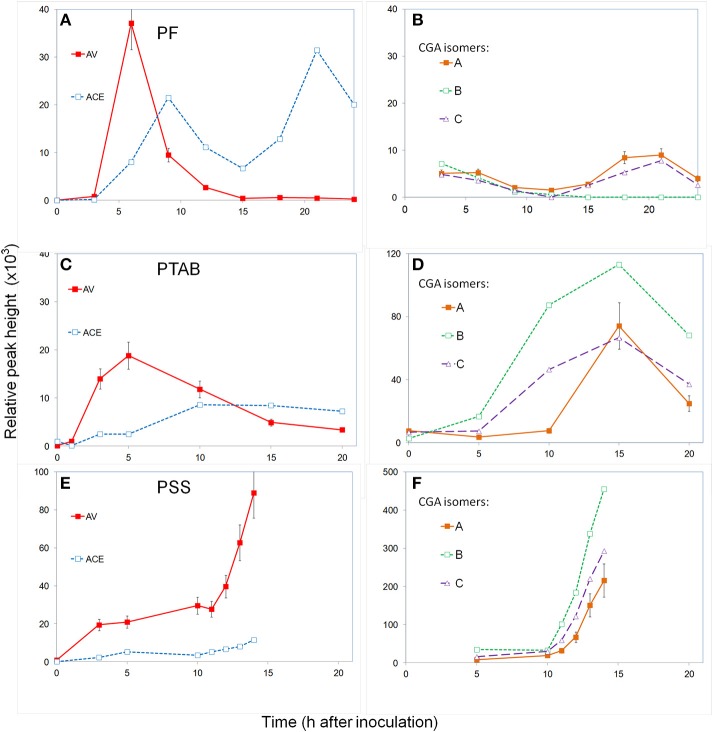
**Time course monitoring apoplast phenolics in tobacco inoculated with ***P. fluorescens*** (A,B), *P.s. tabaci* (C,D), and *P.s. syringae* (E,F)**. The leaf segments were inoculated with 10^8^ CFU ml^−1^ and the apoplast fluid extracted periodically and analyzed for phenolic content using UPLC/UV/MS. Hydroxycinnamic acids **(A,C,E)** and chlorogenic acid isomers **(B,D,F)**. See Materials and Methods for further details; AV (acetovanillone); ACE (acetosyringone); CGA (chlorogenic acid isomers).

Leaves were inoculated with 10^8^ CFU ml^−1^ and the apoplastic fluid was monitored every 3 h for a 24 h period. The experiment was repeated two times. No visible symptoms developed over a 6-day observation period. The level of the two major acetophenones, AV, and ACE, increased after 3 h and peaked by 6–9 h (Figure 4A). The AV level then decreased and remained at baseline by 15 h. The ACE level decreased but after 15 h it peaked again at 24 h. The chlorogenic acid isomers, CGA-A, CGA-B, and CGA-C, remained relatively low during the 24 h period (Figure 4B).

#### *P.s. tabaci*, a virulent pathogen

Leaves were inoculated with 10^8^ CFU ml^−1^ and the apoplastic fluid was monitored for a 20 h period. The experiment was repeated three times. Initially, symptoms of slight water-soaking appeared only at the inoculation site where the barrel of the syringe was pressed gently against the leaf. No visible symptoms occurred until the 2nd day when chlorosis appeared at the edge and beyond the inoculated area followed by increased necrosis over the next 3 days. After 10–15 h the apoplastic fluid appeared slightly brown. The levels of induced AV increased and peaked at the 5 h sampling and then decreased gradually during the remaining period (Figure 4C), similar to the PF treatment. The induced ACE levels increased slowly over the first 10 h period and remained at this relatively low level for the remainder of the 20 h. The CGA isomers remained low during the first 5 h (Figure 4D), but by 15 h they had increased to over 10 times the levels in PF treatments. Isomer B, which was barely detectable in PF treatments, reached its highest level at 15 h and then decreased.

#### *P.s. syringae*, an HR-causing avirulent pathogen

Leaves were inoculated with 10^8^ CFU ml^−1^ and the apoplastic fluid was monitored hourly from 5 to 14 h, after which the tissue was deteriorating and could not be sampled. The experiment was repeated three times. Visible symptoms began after 13 h with increased water-soaking and limp tissue and darkening of tissue appearance and apoplast fluid. Induced AV levels increased by 5 h but unlike other treatments, it maintained this concentration until about 11 h and then increased three to four-fold by 14 h when the last sample was able to be taken (Figure 4E). ACE levels remained relatively low compared to other treatments over the first 10 h and then increased somewhat. The three chlorogenic acid isomers started out at low levels but increased dramatically after 10 h, nearly 100-fold (Figure 4F).

## Discussion

The results of this study demonstrate that the infiltration-centrifugation technique can be used to monitor the composition of apoplast phenolics in tobacco leaves during bacterial inoculation. Leaf age, or position from the top of the plant, was found to have a substantial effect on the composition of phenolics induced by PF (Figure 2). This was compensated for by using only leaves of a certain age on nearly identical plants. Inoculum concentration was another important factor and was nearly directly proportional to the levels of AV and ACE induced by PF (Figure 3). Our initial concerns about bacterial interference with the water infiltration technique proved not to be a problem. The main concern was that after several hours the inoculated bacteria, or newly produced polysaccharides, may in some way block the vacuum infiltration process. However, the amount of water taken up during vacuum infiltration by water-treated and bacteria-treated tissue was always similar, suggesting no blockage.

The induction of phenolics in the plant apoplast by bacteria is a phenomenon similar to that found in cell suspension studies (Baker et al., 2005b, 2009). There appears to be a unique dynamic that determines which phenolics, the magnitiude, and kinetics, depending on the type of plant/bacterial interaction, i.e., saprophytic, virulent, or avirulent. During the 5–10 h after inoculation, the levels of the acetophenones, ACE, and AV, responded differently but may be linked by their biosynthetic pathway (Figures 4A,C,E). Initial evidence suggests AV (3′-methoxy-4′-hydroxyacetophenone) can be formed from ferulic acid through a CoA-dependent pathway (Oh et al., 2010). AV then gives rise to ACE (3′,5′-dimethoxy-4′-hydroxyacetophenone) through O-methylation with S-adenosyl-methionine (Pechanova et al., 2010). AV increased first by 5 h in all treatments reaching the highest levels with *P. fluorescens*. After peaking, AV decreases in saprophytic *P. fluorescens* and virulent *tabaci* treatments, while it increases dramatically after 10 h in the avirulent *P. s. syringae* treatment. The levels of ACE generally lagged behind AV during the first 5–10 h in all treatments. The levels of ACE were also highest in the saprophytic PF interaction, peaking around 9 h and again by 20 h while the level of AV stayed nearly undetectable. The levels of ACE in the virulent PTAB interaction increased slowly to a relatively low level during the first 10 h and remained there for the remainder of time, during which time AV had decreased but did not disappear. The level of ACE in the avirulent *P.s*. pv. *syringae* interaction was similar to that of PTAB during the first 11 h after which it slightly increased reaching prior to leaf collapse. It is during this period that redox reactions are taking place and can have additional effects on these phenolics. The two hydroxyacetophenones responded differently but possibly in a linked manner: AV always appeared first and whenever ACE increased, AV was often decreasing or did not accumulate; this would be consistent with AV being a precursor for ACE.

Inoculation of tobacco with *P. tabaci* resulted in very high levels of chlorogenic acids (Figure 4D). We did not notice these compounds in our characterization studies with *P. fluorescens* because their levels were so low. These secondary metabolites are esters formed by quinic acid and a hydroxycinnamic acid (caffeic, ferulic, or p-coumaric acid). These metabolites have been found throughout the plant kingdom and possess antioxidant activity, which has been found to improve plant defense and biomedical properties (López-Gresa et al., 2011; Upadhyay and Mohan Rao, 2013; Döring and Petersen, 2014; Mhlongo et al., 2014; Ncube et al., 2014). Previous studies, generally based on whole tissue extracts, have related increases in chlorogenic phenolics to disease resistance or possible roles in counteracting pathogen enzymes and toxins (Bostock et al., 1999; López-Gresa et al., 2011; Villarino et al., 2011; Kröner et al., 2012; Hammerschmidt, 2014; Wojciechowska et al., 2014; Li et al., 2015).

The rapid appearance and magnitude of the CGAs in both pathogen treatments, PTAB and PSS, were much different from the patterns in PF treated plants (Figures 4B,D,E). We had not observed such high magnitudes of CGAs in apoplasts of viroid-infected tomato (Baker et al., 2010), nor extracellular fluid of tobacco or potato (Baker et al., 2005b, 2009). There was a possibility that these CGA peaks might be an artifact caused by leakage from the symplast during centrifugation. We carried out some preliminary experiments to check this possibility (data not shown). Extractions of whole leaf panels with acidified water did find high levels of the three CGA isomers, ~350 μM indicating that the symplast did have preexisting high levels of these metabolites, while the untreated apoplast did not. The levels CGA in apoplast extracts from in PTAB and PSS treatments were in the range of 5 μM and 50 μM, respectively. To test the effect of centrifugal force we then applied full and 1/4 strength during apoplast extraction of PTAB 15 h-treated panels from the same leaf. It was apparent by observing the leaves after centrifugation and the amount of fluid recovered, that the lower centrifugal force was not as efficient for complete recovery. However, analysis of these two samples showed nearly identical chromatograms, with peak heights in all of the major and minor phenolics the same. Therefore, preliminary results suggest this sudden increase in CGA isomers was not directly related to the recovery technique, but may be related to an alteration of the apoplast/symplast barrier by the pathogen interaction, allowing leakage to occur. Further examination will be needed to confirm this hypothesis.

This study clearly demonstrated that apoplastic phenolics are induced in tobacco leaves inoculated with different bacteria. In addition, the dynamics of the induced phenolics appeared different depending on the plant/bacteria interaction saprophytic, virulent, or avirulent. Further studies with additional species of each interaction as well as other hosts will be needed to help establish this hypothesis. The possible metabolic pathway connection between AV and ACE needs to be examined more carefully as well as connections with other phenolics that were not reported. These studies of the apoplast can help identify critical redox-sensitive metabolites that have bioactive or redox roles at the active site of the plant/bacterial interaction.

### Conflict of interest statement

The authors declare that the research was conducted in the absence of any commercial or financial relationships that could be construed as a potential conflict of interest.
